# A Meta-Analysis of the Influencing Factors for Tracheostomy after Cervical Spinal Cord Injury

**DOI:** 10.1155/2018/5895830

**Published:** 2018-07-12

**Authors:** Yan Wang, Zhiliang Guo, Dehong Fan, Haijiang Lu, Dong Xie, Dahai Zhang, Yongtian Jiang, Pei Li, Haijun Teng

**Affiliations:** ^1^Weifang Medical University, No. 288 Shengli Street, Kuiwen District, Weifang, Shandong 261000, China; ^2^The Second Department of Spine Surgery, No. 89 Hospital of PLA, No. 256 Beigongxi Street, Weicheng District, Weifang, Shandong 261000, China; ^3^Department of Orthopedic Surgery, No. 89 Hospital of PLA, No. 256 Beigongxi Street, Weicheng District, Weifang, Shandong 261000, China

## Abstract

**Background:**

Traumatic cervical spinal cord injury (CSCI) is a common disease that has high complication, disability, and mortality rates and a poor prognosis. Tracheostomy is an important supportive therapy for patients with CSCI. However, a consensus on the predictive factors for tracheostomy after CSCI has not been reached.

**Objective:**

This meta-analysis study assessed the influencing factors for tracheostomy after CSCI.

**Methods:**

We searched for relevant studies on the influencing factors for tracheostomy after CSCI. The extracted data were analyzed using RevMan 5.3 software. We calculated the odds ratio (OR) or mean difference (MD) and 95% confidence intervals (CIs).

**Results:**

Sixteen eligible studies containing 9697 patients with CSCI were selected. The pooled OR (MD) and 95% CI of the influencing factors were as follows: age (mean ± SD): -0.98 (-4.00 to 2.03), advanced age: 1.93 (0.80 to 4.63), sex (male): 1.29 (1.12 to 1.49), American Spinal Injury Association Impairment Scale (AIS) A grade: 7.79 (5.28 to 11.50), AIS B grade: 1.15 (1.13 to 2.02), AIS C grade: 0.28 (0.20 to 0.41), AIS D grade: 0.04 (0.02 to 0.09), neurological level of injury (upper CSCI): 2.36 (1.51 to 3.68), injury severity score (ISS): 8.97 (8.11 to 9.82), Glasgow Coma Scale (GCS) score ≤8: 6.03 (2.19 to 16.61), thoracic injury: 1.78 (1.55 to 2.04), brain injury: 0.96 (0.55 to 1.69), respiratory complications: 5.97 (4.03 to 8.86), smoking history: 1.45 (0.99 to 2.13), traffic accident injury: 1.27 (0.92 to 1.74), and fall injury: 0.72 (0.52 to 1.01).

**Conclusions:**

The current evidence shows that male sex, AIS A grade, AIS B grade, neurological level of injury (upper CSCI), high ISS, GCS≤8, thoracic injury, and respiratory complications are risk factors for tracheostomy after CSCI, and AIS C grade and AIS D grade are protective factors. This study will allow us to use these factors for tracheostomy decisions and ultimately optimize airway management in patients with CSCI.

## 1. Introduction

Traumatic cervical spinal cord injury (CSCI) is a common disease in orthopedic clinical work; CSCI has high complication, disability, and mortality rates and a poor prognosis [1]. Respiratory failure is the most common lethal factor after traumatic CSCI occurs [1, 2]. Because a tracheostomy can improve the ventilation function, save the patient's life, and improve the treatment efficacy [3–5], it is regarded as an important supportive therapy in patients with CSCI. However, tracheostomy is an invasive operation that may cause tracheal stenosis and stoma infection [6, 7], and an unnecessary and delayed tracheostomy has detrimental effects on patients. Therefore, it is very important to understand the indicators for tracheostomy.

The influencing factors for tracheostomy after CSCI have been widely studied. Among these previous studies, age [8–21], sex [8, 9, 11, 12, 14–16, 19, 20, 22], AIS (American Spinal Injury Association Impairment Scale) [8–22], neurological level of injury (NLI) [8, 9, 11, 12, 14, 16–23], injury seventy score (ISS) [8, 9, 12, 14, 19, 20, 22], Glasgow Coma Scale (GCS) score [8, 11, 14, 15], associated injury [8, 9, 11, 12, 16, 19], respiratory complications [9, 10, 12, 13, 19], smoking history [9, 16, 19, 21], and other factors have been reported. However, a consensus has not been reached due to the differences among these previous results. Thus, the predictive indexes for tracheostomy after CSCI remain unclear and controversial [9, 24].

In this study, reports on the influencing factors for tracheostomy after CSCI were retrieved and comprehensively and quantitatively analyzed. We hope this study contributes to the evidence-based information for respiratory management in patients with CSCI.

## 2. Materials and Methods

### 2.1. Literature Sources

The review process was conducted in accordance with the PRISMA (Preferred Reporting Items for Systematic Reviews and Meta-Analyses) guidelines. The PRISMA checklist is shown in Supplementary Table 1. We searched for studies in the PubMed, EMBASE, Web of Science, Cochrane Library, Chinese Biological Medical Literature (CBM), China National Knowledge Infrastructure (CNKI), and Chinese Wanfang and Chongqing VIP databases. The last search date was January 11, 2018.

### 2.2. Search Strategy and Literature Selection

The search terms used were “cervical spinal cord injury” or “spinal cord injury” and “tracheostomy” and “influencing factors” or “risk factors” or “predictors”. The studies returned were carefully screened by two independent reviewers according to the designed inclusion and exclusion criteria. Briefly, the title and abstract of each article were evaluated to eliminate irrelevant or duplicate studies; then, the full text of the remaining papers was further screened. Disagreements between the reviewers were resolved by discussion.

### 2.3. Inclusion and Exclusion Criteria

Our inclusion criteria were as follows: (1) the study objects were patients with complete or incomplete traumatic CSCI; (2) the study had two groups: a tracheostomy group and a nontracheostomy group; (3) the study provided the original data or odds ratio (OR) and 95% confidence intervals (95% CIs); (4) the definition and quantification of the influencing factors were generally consistent; (5) for the duplicate studies, only the most recently published one was included.

Our exclusion criteria were as follows: (1) the study was an abstract, review, or case report; (2) the study focused on non-CSCI factors; (3) the full text of the study was not available.

### 2.4. Data Extraction

Information, including the title, first author, publication year, authors' countries, patient number, tracheostomy rate, original data, OR value, and 95% CI, was extracted by two independent reviewers. Disagreements between the reviewers were resolved by discussion. If the original data were not complete, we contacted the corresponding author to obtain adequate data if possible.

### 2.5. Quality Evaluation

The quality of the included studies was evaluated using the Newcastle-Ottawa Scale (NOS) as recommended by the International Cochrane Collaboration Network. The scale consists of three parts: (1) selection (is the case definition adequate?; representativeness of the cases, selection of controls, and definition of controls; these parameters comprised 4 entries, and each entry value was 1 point); (2) comparability (comparability of the cases and controls on the basis of the design or analysis; this parameter comprised one entry, and each entry value was 2 points); (3) exposure (ascertainment of exposure; the same ascertainment method for cases and controls and nonresponse rate; these factors comprised three entries, and each entry value was 1 point). Zero to 3 points were designated as low literature quality, 4 to 6 points were designated as medium literature quality, and 7 to 9 points were designated as high literature quality.

### 2.6. Statistical Methods

Statistical analyses were performed using RevMan 5.3 software provided by the Cochrane Collaboration. Continuous variables are expressed as the mean difference (MD) and related 95% CI, whereas dichotomous variables are expressed as the OR and related 95% CI for the effects analysis. If the original study did not provide original case-control data, the OR and 95% CI were extracted directly. The heterogeneity between the results of the study was analyzed by the chi-square test, and the test level was *α*=0.1. If there was no significant heterogeneity among the results (P≥0.1, I^2^≤50%), a fixed-effects model was used for data analysis. If there was significant heterogeneity among the results (P<0.1, I^2^>50%), a random effects model was used for data analysis, and the subgroup analysis and sensitivity analysis were further used to analyze the heterogeneity. The results stability was tested with a sensitivity analysis. Publication bias was evaluated by funnel plot if the included studies were more than 10.

## 3. Results

### 3.1. Literature Information

**Figure 1:**
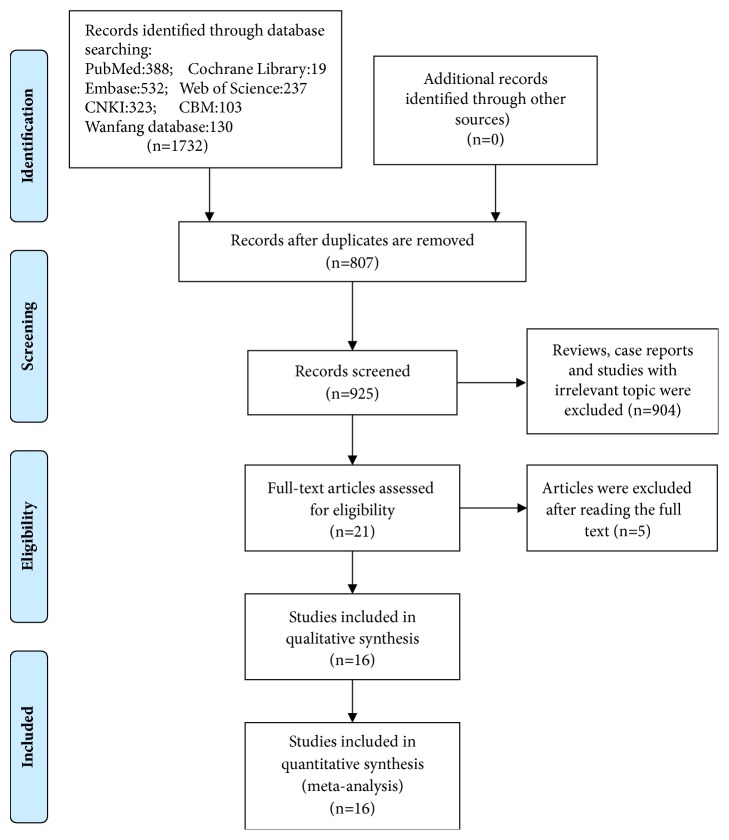
A flow chart of the study selection.

A total of 1732 studies were retrieved from the databases. A description of the literature retrieval and selection process is shown in Figure 1. After selection, 807 repetitive articles and 904 reviews, case reports, and studies on irrelevant topics were excluded. Among the remaining reports, 5 papers were excluded after reading the full text: 2 papers had a large number of patients with thoracic vertebral injury; one paper did not have a nontracheostomy control group; one paper included patients with only complete CSCI; and one paper did not have complete data, and we failed to contact the author. Finally, a total of 16 eligible papers (11 in English and 5 in Chinese) were included. All the eligible papers were case-control studies. A total of 9697 patients with CSCI were included in this meta-analysis; 1773 of the patients underwent tracheostomy, and the tracheostomy rate was estimated to be 18.3%. The study characteristics are shown in Table 1. The literature quality evaluated by the Newcastle-Ottawa Scale (NOS) showed that all studies scored at least 7 points, which indicates high literature quality (Table 2).

### 3.2. Meta-Analysis of Each Common Influencing Factor

For the reported influencing factors, there were at least four eligible studies that focused on age, sex, NLI, AIS, GCS, ISS, associated injury, respiratory complications, smoking history, and injury mechanism. The details of these influencing factors are presented in Table 1. However, there were no more than 2 studies that focused on the other influencing factors, such as cervical fracture or dislocation, vital capacity, forced vital capacity, abdominal injury, and blood pressure; thus, a meta-analysis was not performed on these influencing factors.

### 3.3. Age

In the analysis of eleven studies (7342 patients) using the continuous variable method, significant heterogeneity among these studies (I^2^=73%, p<0.0001) was found, and the results showed that age was not an influencing factor for tracheostomy (MD=-0.98, 95%CI: -4.00 to 2.03; p=0.52, random effects model, Supplementary Fig 1). In the analysis of 6 studies (6833 patients) using the dichotomous variable method (advanced age and nonadvanced age), significant heterogeneity among these studies (I^2^=88%, p<0.00001) was found, and the results showed that advanced age was not an influencing factor for tracheostomy (OR=1.93, 95% CI: 0.80 to 4.63; p=0.14, random effects model, Supplementary Fig 2).

### 3.4. Sex

Ten studies (7092 patients) focused on patient sex. There was no significant heterogeneity among these studies (I^2^=0%, p=0.59). The results showed that sex (male) was an influencing factor for tracheostomy (OR=1.29, 95% CI: 1.12 to 1.49; p=0.0004, random effects model, Supplementary Fig 3).

### 3.5. AIS

**Figure 2:**
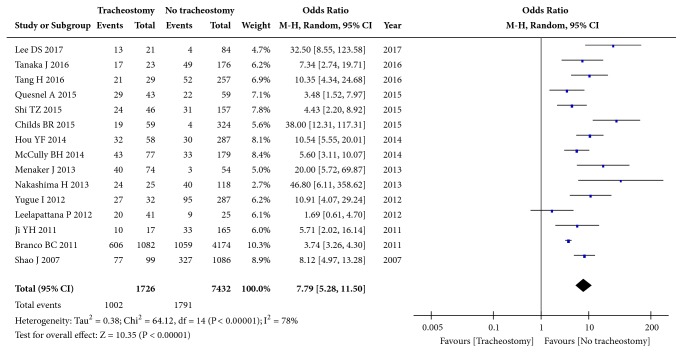
Forest plot of the meta-analysis of AIS A grade in patients with tracheostomy after cervical spinal cord injury (CSCI).

**Figure 3:**
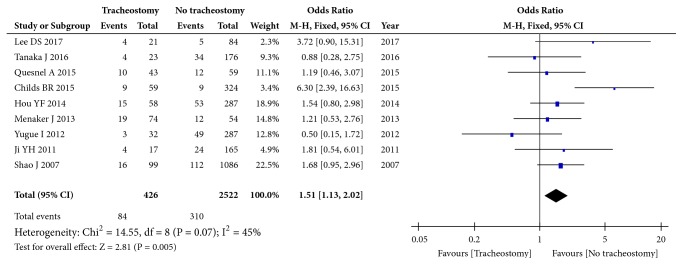
Forest plot of the meta-analysis of AIS B grade in patients with tracheostomy after cervical spinal cord injury (CSCI).

Fifteen studies (9158 patients) focused on AIS. Significant heterogeneity was observed for AIS A grade (I^2^=78%, p<0.00001), and the pooled OR was 7.79 (95% CI: 5.28 to 11.50; p<0.00001, random effects model, Figure 2). Moderate heterogeneity was observed for AIS B grade (I^2^=45%, p=0.07), and the pooled OR was 1.15 (95% CI: 1.13 to 2.02; p=0.005, fixed effects model, Figure 3). No significant heterogeneity was observed for AIS C grade (I^2^=0%, p=0.45), and the pooled OR was 0.28 (95% CI: 0.20 to 0.41; p<0.00001, fixed effects model, Supplementary Fig 4). No significant heterogeneity was observed for AIS D grade (I^2^=0%, p=0.73), and the pooled OR was 0.04 (95% CI: 0.02 to 0.09; p<0.00001, fixed effects model, Supplementary Fig 5). Collectively, these results show that AIS A grade and AIS B grade are risk factors, while AIS C grade and AIS D grade are protective factors.

### 3.6. NLI

Thirteen studies (7822 patients) focused on NLI (upper CSCI). Significant heterogeneity among these studies was observed (I^2^=81%, p<0.00001), and the pooled OR was 2.36 (95% CI: 1.51 to 3.68; p=0.0002, random effects model, Supplementary Fig 6). Because of the different NLI classifications among the reports, we performed a subgroup analysis using the dichotomous variable method (NLI≥C4 versus NLI<C4 or NLI≥C5 versus NLI<C5). For the subgroup analysis of NLI≥C4, obvious heterogeneity was observed (I^2^=83%, p=0.00001), and the pooled OR was 2.54 (95% CI: 1.52 to 4.25; p=0.0004, random effects model, Supplementary Fig 6). For the subgroup analysis of NLI≥C5, obvious heterogeneity was also observed (I^2^=82%, p=0.02), and the pooled OR was 1.83 (95% CI: 0.43 to 7.76; p=0.41, random effects model, Supplementary Fig 6).

### 3.7. ISS

**Figure 4:**
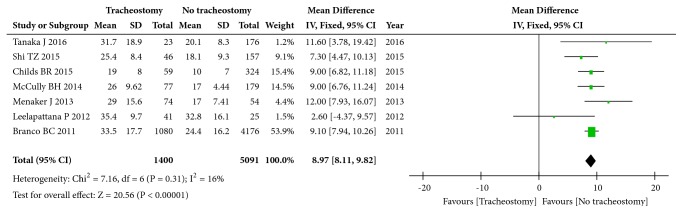
Forest plot of the meta-analysis of ISS in patients with tracheostomy after cervical spinal cord injury (CSCI).

Seven studies (6073 patients) with continuous variables focused on ISS. There was mild heterogeneity among these studies (I^2^=16%, p=0.31), and the pooled MD was 8.97 (95%CI: 8.11 to 9.82; p<0.00001, fixed effects model, Figure 4), indicating that a high ISS is an influencing factor for tracheotomy after CSCI.

### 3.8. GCS

**Figure 5:**
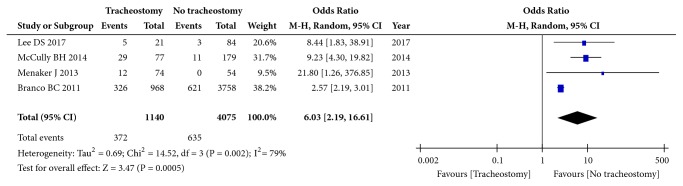
Forest plot of the meta-analysis of GCS ≤ 8 in patients with tracheostomy after cervical spinal cord injury (CSCI).

Four studies (5215 patients) focused on GCS using the dichotomous variable method (GCS≥8 vs. GCS<8). There was significant heterogeneity among these studies (I^2^=79%, p=0.002), and the pooled OR was 6.03 (95% CI: 2.19 to 16.61; p=0.0005, random effects model, Figure 5), suggesting that GCS≥8 is an influencing factor for tracheotomy after CSCI.

### 3.9. Associated Injury

**Figure 6:**
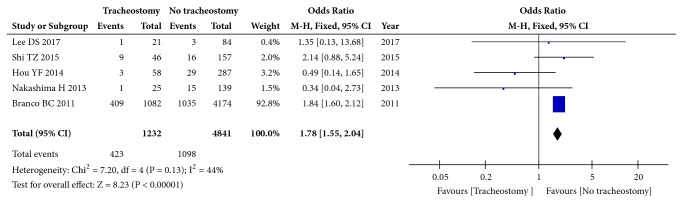
Forest plot of the meta-analysis of thoracic injury in patients with tracheostomy after cervical spinal cord injury (CSCI).

Five studies (6073 patients) focused on the associated injury using the dichotomous variable method (with thoracic injury versus without thoracic injury; with brain injury versus without brain injury). The analysis of thoracic injury revealed moderate heterogeneity (I^2^=44%, p=0.13), and the pooled OR was 1.78 (95% CI: 1.55 to 2.04; p<0.00001, fixed effects model, Figure 6). The analysis of brain injury revealed no heterogeneity (I^2^=0%, p=0.49), and the pooled OR was 0.96 (95%CI: 0.55 to 1.69; p=0.89, fixed effects model, Supplementary Fig 7). Together, these results suggest that thoracic injury but not brain injury is an influencing factor for tracheotomy after CSCI.

### 3.10. Respiratory Complications

**Figure 7:**
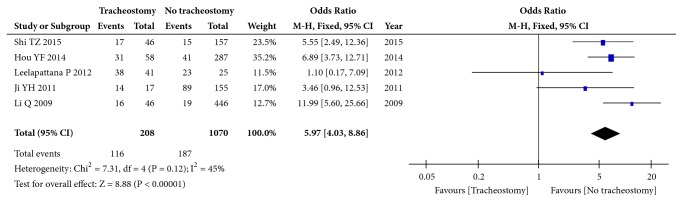
Forest plot of the meta-analysis of respiratory complications in patients with tracheostomy after cervical spinal cord injury (CSCI).

Five studies (1278 patients) focused on respiratory complications. The results showed that there was moderate heterogeneity among these studies (I^2^=45%, p=0.12), and the pooled OR among these studies was 5.97 (95%CI: 4.03 to 8.86; p<0.00001, fixed effects model, Figure 7), suggesting that respiratory complications are an influencing factor for tracheotomy after CSCI.

### 3.11. Smoking History

Four studies (1031 patients) focused on smoking history. The results showed that there was moderate heterogeneity among these studies (I^2^=46%, p=0.14), and the pooled OR was 1.45 (95% CI: 0.99 to 2.13; p=0.06, fixed effects model, Supplementary Fig 8). There was no statistically significant difference.

### 3.12. Injury Mechanism

We performed a meta-analysis on two types of injury mechanisms: traffic accidents and fall injuries. Five studies (1027 patients) focused on traffic accidents. There was no significant heterogeneity among these studies (I^2^=0%, p=0.78), and the pooled OR was 1.27 (95%CI: 0.92 to 1.74; p=0.14, fixed effects model, Supplementary Fig 9). Four studies (922 patients) focused on fall injuries. There was no significant heterogeneity among these studies (I^2^=0%, p=0.81), and the pooled OR was 0.72 (95% CI 0.52 to 1.01; p=0.06, fixed effects model, Supplementary Fig 10). These results suggest that both traffic accidents and fall injuries are not influencing factors for tracheotomy after CSCI.

### 3.13. Sensitivity Analysis

For the influencing factors of age, sex, NLI, AIS A grade, AIS C grade, AIS D grade, ISS, brain injury, respiratory complications, traffic accident injury, and fall injury, there were no significant changes in heterogeneity or pooled effect estimates. However, for GCS≤8, removing Branco's study reduced the I^2^ value to 0% and changed the pooled OR value to 10.19 (95% CI 5.17 to 20.08; p<0.00001, fixed effects model). For thoracic injury and AIS B grade, removing Hou YF's study and Childs BR's study, respectively, reduced the I^2^ values to 0% and to 45% but did not change the pooled OR values.

### 3.14. Assessment of Publication Bias

The funnel plots for age (mean±SD), AIS A grade, and NLI were symmetrical and concentrated, suggesting that there is little possibility of publication bias (Supplementary Fig 11-13). The funnel plot for sex (male) was poorly symmetrical and scattered, suggesting that there is a certain amount of publication bias (Supplementary Fig 14).

## 4. Discussion

Respiratory complications are the most common complication and the most common cause of death in patients with CSCI [1, 3, 12, 25, 26]. Airway management, including mechanical ventilation, tracheal intubation, and tracheostomy, is particularly important in patients with severe CSCI, especially patients with respiratory failure [27]. It is reported that the tracheostomy rate ranges from 8.4% to 62.1% in patients with CSCI [8–23]. Although many studies have investigated the factors that affect tracheostomy after CSCI, the results are different, and a consensus on the predictive factors for tracheostomy has not yet been reached [9, 24]. This study is the first to perform a meta-analysis on various reported factors that influence tracheostomy. Our results showed that male sex, AIS A grade, AIS B grade, NLI (upper CSCI), high ISS, GCS≤8, thoracic injury, and respiratory complications were risk factors for tracheostomy after CSCI, whereas AIS C grade and AIS D grade were protective factors.

Our results showed that age is not an influencing factor for tracheostomy after CSCI. Velmahos et al. [28] reported that there was no relationship between patient age and tracheal intubation or respiratory complications in patients with CSCI. Our study also found that tracheostomy was not related to the patient's age. The results showed that male sex is an influencing factor for tracheostomy after CSCI (OR=1.29). As previously reported, male patients have a high rate of smoking, which can affect the function of ventilation and reduce the FEV1 and FEV1/FVC [29]. Other scholars have indicated that smoking often causes excessive airway secretions and airway inflammation [30, 31] and that the increased secretion of bronchial mucus ultimately leads to pneumonia in patients with CSCI [16]. However, our meta-analysis results showed that smoking was not an influencing factor for tracheostomy after CSCI (OR=1.45, 95% CI: 0.99 to 2.13, p=0.06). On the other hand, we also noticed that the results were very close to reaching statistical significance. Since only four eligible articles focused on smoking, more evidence-based information is needed to determine whether smoking is an influencing factor for tracheostomy after CSCI.

The present results showed that AIS A grade and AIS B grade were influencing factors for tracheostomy after CSCI. Importantly, AIS A grade with an OR value of 7.79 was indicated to have a significant role in determining tracheostomy performance. In line with our results, several previous reports support the idea that AIS A grade is an independent predictor for tracheotomy with multivariate logistic regression analyses [8, 9, 11, 13, 16, 19, 21, 22]. Lee et al. proposed that AIS A grade was the most important predictor of tracheotomy for patients with CSCI [11]. Childs et al. showed that AIS A grade was an early indicator for tracheotomy in patients with CSCI [22]. Patients with AIS A grade have a high tracheostomy rate; the low incidences of irreversible injury resulting from tracheostomy and the benefits of early tracheostomy indicate that tracheostomy should be performed in all patients with AIS A grade [22].

There are considerable controversies surrounding the influence of NLI on tracheotomy. Because the diaphragm, which is mainly responsible for respiratory function, is primarily innervated by C4 and partially innervated by C3 and C5, many researchers believe that upper CSCI is an influential factor for tracheotomy. However, several research teams, such as Branco et al. [8], McCully et al. [14], Nakashima et al. [16], and Childs et al. [22], demonstrated that there was no significant correlation between NLI (upper CSCI) and tracheotomy. Childs et al. noted that the auxiliary muscles important for breathing are affected by lower cervical cord injury and that though lower CSCI does not directly affect the innervation of the diaphragm, chest wall and abdominal muscle paralysis can lead to dyspnea [22]. Patients with incomplete CSCI, regardless of NLI, are less likely to require a tracheostomy [14]. In this study, we found that upper CSCI (OR=2.36) was an influencing factor for tracheotomy, and a subgroup analysis showed that there was a statistically significant correlation between NLI≥C4 and tracheotomy, while there was no significant correlation between NLI≥C5 and tracheotomy. This result may be because many patients with C5 spinal cord injury do not require tracheotomy. We thus considered NLI≥C4 as a predictor of tracheotomy. Of note, six studies [12, 17, 18, 20–22] (1260 patients) focused on NLI using the ordered categorical variable method (C1, C2, C3, C4, etc.). To facilitate statistical calculations, we converted the ordered categorical variable of NLI into a dichotomous variable. This conversion process can result in missing information that may influence the results.

This study showed that a high ISS and GCS≤8 are related to tracheostomy in patients with cervical cord injury. Patients who have a high ISS and low GCS (GCS≤8) need a tracheostomy and treatments aimed at respiratory complications [8, 14, 15]. However, Velomahos et al. demonstrated that it is difficult to accurately measure the ISS because the ISS is easily alterable in the acute phase of trauma (within 2 days after injury), and they did not recommend it as a predictor for tracheostomy [28]. In future studies, accurately calculating the ISS is a problem that needs to be explored.

This study also found that thoracic injury and respiratory complications are influencing factors for tracheostomy after CSCI. Complications, such as pneumonia and pleural effusion, can further limit the damaged respiratory function and aggravate hypoxia and the resulting secondary spinal cord injury [9]. Additionally, complications, such as pneumonia and atelectasis, were confirmed to be related to prolonged mechanical ventilation, which increases tracheostomy rates [32]. Leelapattana et al. [12] also showed that an oxygenation index (PaO2/FiO2) <300 was associated with tracheostomy after 3 days of mechanical ventilation in patients with CSCI. However, the mechanism of injury was not found to be associated with tracheostomy in this meta-analysis.

In this study, the literature was screened by using rigorous inclusion and exclusion criteria, and the literature quality was evaluated with the NOS. Sensitivity analyses did not change the direction or statistical significance of any pooled effect estimates but did change the level of heterogeneity of several factors (GCS≤8, thoracic injury and AIS B grade). Several limitations exist in this study. First, publication bias could not be avoided in this study. To avoid publication bias as much as possible, the processes of study selection, data extraction, and quality assessment were performed by two independent researchers. Second, the eligible studies were retrospective case-control studies, which are inferior to prospective studies in design rigidity and persuasion. However, the total sample size (n=9697) is acceptable, which can overcome this limitation to some extent. Third, the definition of certain influencing factors is not consistent (i.e., advanced age: Lee et al. [11], McCully et al. [14], and Branco et al. [8] defined advanced age as ≥55 years; Yugue et al. [21] defined advanced age as ≥69 years; and Nakashima et al. [16] defined it as >45 years). Fourth, due to the limited literature and data, some influencing factors with obvious heterogeneity could not be further analyzed with a subgroup analysis to find heterogeneity sources. Fifth, other factors studied, such as spine fracture or dislocation, vital capacity, forced vital capacity, abdominal injury, and blood pressure, could not be analyzed due to the limited studies; thus, other meaningful and valuable influencing factors could have been missed.

## 5. Conclusions

In conclusion, we conducted a meta-analysis on the influencing factors for CSCI. To some extent, this meta-analysis resolves the controversy of the indicators for tracheostomy and provides evidence-based information to guide clinicians in balancing the necessity of tracheostomy or early tracheostomy and the complications of tracheostomy. In future clinical studies, we can use these factors, which include male sex, AIS A grade, AIS B grade, high NLI (upper CSCI), high ISS, GCS≤8, thoracic injury, and respiratory complications, to determine tracheostomy decisions for patients with CSCI.

## Figures and Tables

**Table 1:** Basic characteristics of the included studies.

Author	Publication year	Country	Case number	Tracheostomy rate	Research factors
Lee DS [11]	2017	Korea	105	20.0%	1,2,3,4,6,7,10
Tanaka J [20]	2016	Japan	199	11.6%	1,2,3,4,5
Tang H [23]	2016	China	286	10.1%	3,4
Shi TZ [19]	2015	China	203	27.7%	1,2,3,4,5,7,8,9
Childs BR [22]	2015	USA	383	15.4%	2,3,4,5,10
Quesnel A [17]	2015	France	108	40.7%	1,3,4
Hou YF [9]	2014	China	345	16.8%	1,2,3,4,7,8,9,10
McCully BH (14]	2014	USA	256	30.1%	1,2,3,4,5,6
Menaker J [15]	2013	USA	128	57.8%	1,2,3,5,6,10
Nakashima H [16]	2013	Japan	164	15.2%	1,2,3,4,7,9
Leelapattana P [12]	2012	Canada	66	62.1%	1,2,3,4,5,7,8,10
Yugue I [21]	2012	Japan	319	10.0%	1,3,4,9
Branco BC [8]	2011	USA	5256	20.6%	1,2,3,4,5,6,7
Ji YH [10]	2011	China	182	9.3%	1,3,8
Li Q [13]	2009	China	512	9.0%	1,8
Shao J [18]	2007	China	1185	8.4%	1,3,4

Note: 1: age; 2: sex; 3: AIS (American Spinal Injury Association Impairment Scale); 4: NLI (neurological level of injury); 5: ISS (injury severity score); 6: GCS (Glasgow Coma Scale); 7: associated injury; 8: respiratory complications; 9: smoking history; 10: mechanism of injury. NLI refers to the most caudal segment of the spinal cord with normal sensory and motor functions on both sides of the body. Respiratory complications were defined as pneumonia, complicated pleural effusion, and atelectasis requiring additional oxygen supply. AIS is now based on the International Standards for the Neurological Classification of Spinal Cord Injury (ISNCSCI). Thoracic injury was defined as pneumothorax, hemothorax, or frail chests. The mechanism of injury refers to traffic accidents, falls, or others.

**Table 2:** Literature quality of the included studies.

First author	Selection				Comparability	Exposure			Total score
	I	II	III	IV	V	VI	VII	VIII	
Lee DS [11]	1	1	1	1	2	1	1	1	9
Tanaka J [20]	1	1	1	1	2	1	1	NA	8
Tang H [23]	1	1	1	1	2	1	1	NA	8
Shi TZ [19]	1	1	1	1	2	1	1	NA	8
Childs BR [22]	1	1	1	1	2	1	1	1	9
Quesnel A [17]	1	1	1	1	2	1	1	NA	8
Hou YF [9]	1	1	1	1	2	1	1	NA	8
McCully BH [14]	1	1	1	1	2	1	1	NA	8
Menaker J [15]	1	1	1	1	2	1	1	NA	8
Nakashima H [16]	1	1	1	1	2	1	1	NA	8
Leelapattana P [12]	1	1	1	1	2	1	1	NA	8
Yugue I [21]	1	1	1	1	2	1	1	1	9
Branco BC [8]	1	1	1	1	2	1	1	1	9
Ji YH [10]	1	1	1	1	2	1	1	NA	9
Li Q [13]	1	1	1	1	2	1	1	NA	8
Shao J [18]	NA	1	1	1	2	1	1	NA	7

Note: I: is the case definition adequate?; II: representativeness of the cases; III: selection of controls; IV: definition of controls; V: comparability of cases and controls on the basis of the design or analysis; VI: ascertainment of exposure; VII: same ascertainment method for cases and controls; VIII: nonresponse rate; NA: not available.

## Data Availability

The data supporting this meta-analysis are from previously reported studies, which have been cited. The processed data are available in the forest plot.

## Supplementary Materials

Supplementary 1 Supplementary Table 1: PRISMA 2009 checklist.

Supplementary 2 Supplementary Fig 1: forest plot of the meta-analysis of age (mean ± SD) in patients with tracheostomy after cervical spinal cord injury (CSCI).

Supplementary 3 Supplementary Fig 2: forest plot of the meta-analysis of advanced age in patients with tracheostomy after cervical spinal cord injury (CSCI).

Supplementary 4 Supplementary Fig 3: forest plot of the meta-analysis of sex (male) in patients with tracheostomy after cervical spinal cord injury (CSCI).

Supplementary 5 Supplementary Fig 4: forest plot of the meta-analysis of AIS C grade in patients with tracheostomy after cervical spinal cord injury (CSCI).

Supplementary 6 Supplementary Fig 5: forest plot of the meta-analysis of AIS D grade in patients with tracheostomy after cervical spinal cord injury (CSCI).

Supplementary 7 Supplementary Fig 6: forest plot of the meta-analysis of NLI in patients with tracheostomy after cervical spinal cord injury (CSCI).

Supplementary 8 Supplementary Fig 7: forest plot of the meta-analysis of brain injury in patients with tracheostomy after cervical spinal cord injury (CSCI).

Supplementary 9 Supplementary Fig 8: forest plot of the meta-analysis of smoking history in patients with tracheostomy after cervical spinal cord injury (CSCI).

Supplementary 10 Supplementary Fig 9: forest plot of the meta-analysis of traffic accident injury in patients with tracheostomy after cervical spinal cord injury (CSCI).

Supplementary 11 Supplementary Fig 10: forest plot of the meta-analysis of fall injury in patients with tracheostomy after cervical spinal cord injury (CSCI).

Supplementary 12 Supplementary Fig 11: funnel plot tests for age (mean ± SD).

Supplementary 13 Supplementary Fig 12: funnel plot tests for AIS A grade.

Supplementary 14 Supplementary Fig 13: funnel plot tests for NLI.

Supplementary 15 Supplementary Fig 14: funnel plot tests for sex (male).
