# Supplementary material for: A Meta-Analysis of the Influencing Factors for Tracheostomy after Cervical Spinal Cord Injury
Source: Biomed Res Int. 2018 Jul 12;2018:5895830. doi: 10.1155/2018/5895830 (PMC6077662; doi:10.1155/2018/5895830)
Supplement: Supplementary 1 — Supplementary Table 1: PRISMA 2009 checklist. [file 5895830.f1.docx]

**Supplementary Fig 1:** Forest plot of the meta-analysis of age (mean ± SD) in patients with tracheostomy after cervical spinal cord injury (CSCI).
