# Supplementary material for: A Meta-Analysis of the Influencing Factors for Tracheostomy after Cervical Spinal Cord Injury
Source: Biomed Res Int. 2018 Jul 12;2018:5895830. doi: 10.1155/2018/5895830 (PMC6077662; doi:10.1155/2018/5895830)
Supplement: Supplementary 11 — Supplementary Fig 10: forest plot of the meta-analysis of fall injury in patients with tracheostomy after cervical spinal cord injury (CSCI). [file 5895830.f11.docx]

**Supplementary Fig 11:** Funnel plot tests for age (mean ± SD).
