# Supplementary material for: A Meta-Analysis of the Influencing Factors for Tracheostomy after Cervical Spinal Cord Injury
Source: Biomed Res Int. 2018 Jul 12;2018:5895830. doi: 10.1155/2018/5895830 (PMC6077662; doi:10.1155/2018/5895830)
Supplement: Supplementary 14 — Supplementary Fig 13: funnel plot tests for NLI. [file 5895830.f14.docx]

**Supplementary Fig 14:** Funnel plot tests for sex (male).
